# Casein phosphopeptides drastically increase the secretion of extracellular proteins in *Aspergillus awamori*. Proteomics studies reveal changes in the secretory pathway

**DOI:** 10.1186/1475-2859-11-5

**Published:** 2012-01-10

**Authors:** Katarina Kosalková, Carlos García-Estrada, Carlos Barreiro, Martha G Flórez, Mohammad S Jami, Miguel A Paniagua, Juan F Martín

**Affiliations:** 1 From INBIOTEC, Instituto de Biotecnología de León, Avda. Real n°. 1, Parque Científico de León, 24006 León, Spain; 2 From Área de Microbiología, Departamento de Biología Molecular, Universidad de León, Campus de Vegazana s/n; 24071 León, Spain

**Keywords:** secretory pathways, chymosin, filamentous fungi, casein phosphopeptides, vesicles, extracellular proteins

## Abstract

**Background:**

The secretion of heterologous animal proteins in filamentous fungi is usually limited by bottlenecks in the vesicle-mediated secretory pathway.

**Results:**

Using the secretion of bovine chymosin in *Aspergillus awamori *as a model, we found a drastic increase (40 to 80-fold) in cells grown with casein or casein phosphopeptides (CPPs). CPPs are rich in phosphoserine, but phosphoserine itself did not increase the secretion of chymosin. The stimulatory effect is reduced about 50% using partially dephosphorylated casein and is not exerted by casamino acids. The phosphopeptides effect was not exerted at transcriptional level, but instead, it was clearly observed on the secretion of chymosin by immunodetection analysis. Proteomics studies revealed very interesting metabolic changes in response to phosphopeptides supplementation. The oxidative metabolism was reduced, since enzymes involved in fermentative processes were overrepresented. An oxygen-binding hemoglobin-like protein was overrepresented in the proteome following phosphopeptides addition. Most interestingly, the intracellular pre-protein enzymes, including pre-prochymosin, were depleted (most of them are underrepresented in the intracellular proteome after the addition of CPPs), whereas the extracellular mature form of several of these secretable proteins and cell-wall biosynthetic enzymes was greatly overrepresented in the secretome of phosphopeptides-supplemented cells. Another important 'moonlighting' protein (glyceraldehyde-3-phosphate dehydrogenase), which has been described to have vesicle fusogenic and cytoskeleton formation modulating activities, was clearly overrepresented in phosphopeptides-supplemented cells.

**Conclusions:**

In summary, CPPs cause the reprogramming of cellular metabolism, which leads to massive secretion of extracellular proteins.

## Background

Filamentous fungi are very attractive host organisms for the production of heterologous proteins, since they have several advantages for protein expression compared to bacterial hosts. These advantages include i) the ability to produce large amounts of extracellular proteins, ii) the GRAS status in the food industry of several filamentous fungi such as *Aspergillus niger, Aspergillus awamori, Aspergillus oryzae, Penicillium roqueforti *among others [1,2], iii) rapid growth compared to other eukaryotic cells, iv) the secretion of correctly folded functional proteins and v) post-translational modifications, such as glycosylation. However, the levels of secreted heterologous proteins are often limited by poorly understood bottlenecks in the secretory pathway [3,4]. In many cases, limiting steps in the heterologous protein secretion occur during protein movement through the secretory pathway [5-9]. Particularly, structural or regulatory proteins affecting the traffic through the secretory vesicles are unknown. Therefore, a good understanding of the protein-secretion pathway is needed for the improvement of heterologous proteins production in industrial processes. Proteomics is an excellent tool for this study.

The protein secretion pathway in filamentous fungi is similar to that present in yeasts and higher eukaryotes, but protein secretion is believed to occur mainly at hyphal tips [10,11]. The classical secretory pathway of proteins across membranes starts with the recognition and cleavage of a canonical N-terminal signal peptide (the *pre *sequence). These proteins enter the endoplasmic reticulum (ER), where they are correctly folded and modified (glycosylation, phosphorylation, etc) [9,12] and later reach the Golgi compartment, where proteins can undergo additional modifications, such as changes of the lateral chains of some amino acids, the addition or trimming down of sugars and other types of peptide processing. After this step, proteins are packed in secretory vesicles and directed to the plasma membrane for secretion, or targeted to the vacuole either to become resident proteins or to undergo proteolytic degradation [6,13]. Heterologous proteins might lack some of the features needed to be efficiently recognized as genuine secretory proteins and, therefore, their secretion is more difficult.

Filamentous fungi of industrial interest include *A. niger *[2] and the closely related species *A. awamori*, widely used for the expression of homologous (e.g. glucoamylase) and heterologous proteins, such as human lactoferrin [14], cytokines [15] and proteins for the food industry including bovine chymosin [16,17] or thaumatin [8].

Bovine chymosin, an aspartyl protease extracted from the abomasum of suckling calves is an excellent model protein to study possible bottlenecks in the secretion of heterologous proteins. It is synthesized *in vivo *as preprochymosin and secreted as prochymosin (with a molecular mass of 46 kDa), which is autocatalytically activated to chymosin (molecular mass 35.6 kDa).

In a previous work, a synthetic gene (*chy*) encoding the bovine chymosin was constructed (with codon usage optimized for *Aspergillus*) and expressed in *A. awamori*. The preprochymosin was synthesized in this fungus, with the release of the correct prochymosin that was self-processed yielding the mature chymosin [18]. During the optimization studies of heterologous proteins production in *A. awamori*, we observed that there is a bottleneck in the proper protein folding [12]. Other steps that occur during protein traffic through vesicles may also be limiting. Unfortunately, the vesicle targeting system and the final step in which the secretory vesicles are fused to the plasma membrane and release their content, is largely unknown. This step seems to be activated by fusogenic proteins [19].

We describe in this article that casein-derived phosphopeptides drastically stimulate the secretion of prochymosin. Expression analyses revealed that the stimulatory effect of these phosphopeptides is not exerted at transcriptional level. Proteomics analyses provided evidence of an important role of charged casein phosphopeptides (CPPs) in triggering the traffic of chymosin through the secretory pathway, which leads to the export of this and other extracellular proteins.

## Results

### Effect of casein on extracellular bovine chymosin production using five different recombinant strains

In initial studies, we observed that casein, which is partially hydrolyzed in the *A. awamori *cultures, exerts a strong stimulatory effect on chymosin production.

**Figure 1:**
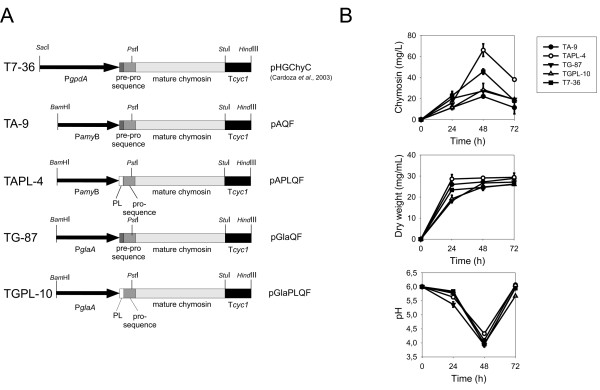
**A. Physical map of the expression cassettes that are present in strains T7-36 (plasmid pHGChyC), TA-9 (pAQF), TAPL-4 (plasmid pAPLQF), TG-87 (pGlaQF) and TGPL-10 (pGLaPLQF)**. The synthetic chymosin gene is flanked by the promoter region of the *gpdA *(in the original strain T7-36), *amyB *or *glaA *genes, which are indicated by a black arrow. Protein secretion signals are indicated as a shaded box (pre sequence of the pre-prochymosin) or an open box (PL, leader peptide of α-amylase or glucoamylase). The T*cyc1 *transcriptional terminator from *S. cerevisiae *is represented as a black box. **B**. Time course of chymosin production, growth and pH profiles of the above-mentioned strains cultured in flasks containing MDF3 medium (including 10 g/L casein). Error bars indicate the standard deviation of three analyses made from three independent cultures.

The stimulatory effect of casein on the secretion of bovine chymosin was studied using five *A. awamori *strains, which contain different expression cassettes for the *chy *gene (Figure 1A). Transformant T7-36 (reference strain), harbours a construction in which the complete pre-pro-*chy *gene is expressed from the constitutive promoter of the *A. nidulans gpdA *gene (P*gpdA*) [18]. The second strain (TA-9) carries a construction in which the pre-pro-*chy *gene is expressed from the *A. oryzae amyB *promoter. In the third strain (TAPL-4), the *chy *gene is also expressed from the *amyB *promoter, but the *pre *sequence of chymosin has been replaced by the leader peptide (21 amino acids) of the *A. oryzae *AmyB protein in such a way that the sequence and all transcriptional signals of the *amyB *gene are retained together with the promoter. The fourth strain (TG-87) carries a construction in which the pre-pro-*chy *gene is expressed from the *A. niger *glucoamylase gene (*glaA*) promoter. The fifth strain (TGPL-10) bears a similar construction, with the exception of the leader peptide (pre sequence), which was that of the *A. niger *glucoamylase instead of that of pre-prochymosin.

In the above-mentioned constructions, transcription of the *chy *gene is terminated with the Tcyc1 (transcriptional terminator of the cytochrome oxidase gene) of *Saccharomyces cerevisiae*.

Production of extracellular chymosin by those strains was calculated in casein-supplemented medium (see below). Results showed that TAPL-4, in which the *chy *gene is expressed from the *A. oryzae amyB *promoter, yielded consistently higher levels of active extracellular chymosin than the other strains. There were no significant differences in the growth kinetics and the pH profiles among the five strains, except for the growth of TG-87, which was always a bit slower than the other strains (Figure 1B).

### The secretion of chymosin is drastically increased upon the addition of casein to the culture médium

**Figure 2:**
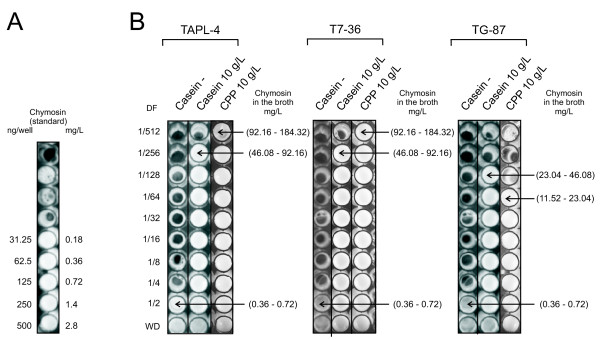
**Effect of casein and casein phosphopeptides addition on chymosin production**. **A**. Serial dilutions (1/2) of a pure preparation of chymosin (500 ng in 175 μl). **B**. Culture supernatants from strains TAPL-4, T7-36 and TG-87 cultured in the absence (-) or presence of either casein or phosphopeptides (CPP) were serially diluted from 1/2 to 1/512 (DF). No dilution is denoted as WD. The number of wells where milk clotting takes place (white precipitate) is proportional to the concentration of chymosin that is present in the culture supernatant. The chymosin concentration range that is present in those wells with positive clotting, has been determined using the data obtained from panel A as indicated in the Methods section.

Experiments showed that the addition of casein (10 g/L) to the culture medium is essential for chymosin secretion. Since the medium contains ammonium sulfate as nitrogen source, growth was not significantly affected by the addition of casein. Only between 0.36-0.72 mg/L of chymosin were secreted in the absence of casein, whereas those cultures supplemented with casein secreted chymosin at a concentration range from 23.04-46.08 mg/L (TG-87 strain) or 46.08-92.16 mg/L (TAPL-4 and T7-36 strains) after 48 h of culture (Figure 2B). The same results were obtained with the addition of 5 g/L casein.

In order to test whether the casein effect was due to the casein protein itself or to the amino acids of this protein, the effect of casein was compared to that of casamino acids (casein hydrolysates) (Bacto™) and NZ Amino A (Sigma). Results indicated that strains TG-87 and TAPL-4 supplemented with casamino acids failed to increase the secretion of chymosin above 0.36-0.72 mg/L, although the medium with NZ Amino A gave rise to a slightly higher secretion (Additional File 1). Since casamino acids did not exert the stimulatory action, this effect seems to be due to the phosphopeptides existing in casein.

### Casein phosphopeptides stimulate chymosin secretion

Casein is partially hydrolyzed to peptides by the proteolytic activities of *A. awamori*. An important fraction of casein peptides are the phosphopeptides. Evidence obtained with intestinal membranes indicates that CPPs trigger cytokine secretion [20]. Furthermore, CPPs elicited IL-6 cytokine release from cultured intestinal epithelial cells [21]. A similar secretion-enhancing mechanism may explain the stimulation of chymosin production in *A. awamori*. To test the effect of CPPs, cultures of *A. awamori *T7-36, TAPL-4 and TG-87 were grown in medium containing 10 g/L CPPs (commercial CE90CPP obtained from DMV International).

Growth parameters remained similar with or without CPPs. However, chymosin production reached the same levels (or even higher with strains TAPL-4 and T7-36) as those observed after casein addition (Figure 2B). The same results were obtained with the addition of 5 g/L CPPs. Therefore, CPPs also stimulate the production of extracellular chymosin. This effect seems to be slightly stronger than that provided by casein.

Since CPPs are rich in phosphoserine (see Discussion), experiments were carried out as described above, but including 10 g/L phosphoserine (Sigma-Aldrich). As it can be seen in Additional File 1, chymosin secretion levels remained similar to those obtained with the control (unsupplemented) samples, indicating that phosphoserine itself is not responsible for the positive stimulatory effect exerted by CPPs on chymosin production.

### Physiological parameters affected by the addition of either casein or CPPs

**Figure 3:**
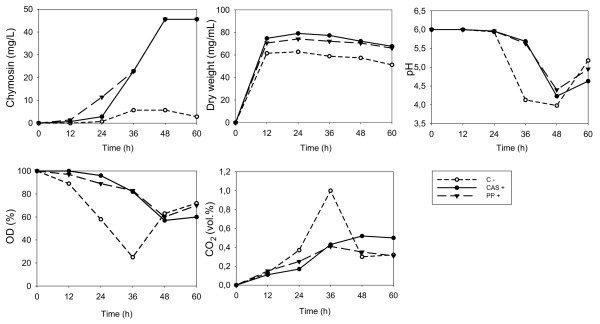
**Physiological parameters obtained after the fermentation of the TAPL-4 strain in three identical 5-L Biostat B (Braun, Germany) fermentors**. OD (%): percentage of dissolved O_2_; CO_2 _(vol.%): percentage of released CO_2_. Casein (CAS +) or CPPs (PP +) were added at a concentration of 10 g/L. The reference condition (C-) did not contain casein or CPPs.

In order to evaluate whether the effect caused by the addition of either casein or CPPs is an indirect consequence of adding a complex proteinaceous substrate and energy source, fermentations were carried out with the above-mentioned strains in a Biostat B (Braun, Germany) fermentor (5 L). Parameters such as dry weight (mg/mL), pH, dissolved O_2 _and the CO_2 _released were analyzed. All strains provided similar results and therefore, we chose the TAPL-4 strain as a model. In general, casein and CPPs provided the same response (Figure 3). Under control conditions (without the addition of either casein or CPPs), TAPL-4 growth showed the same kinetic as in the presence of casein or CPPs, although biomass levels were slightly lower. Another interesting result was the lower levels of dissolved O_2 _(with the subsequent increase in the CO_2 _levels that were released) provided by the reference condition. The decrease in pH was also faster without the addition of casein or CPPs (Figure 3). These results indicate that casein and CPPs affect several cellular physiological aspects, specially those related to the use of oxygen (see Discussion).

### Dephosphorylation of casein decreases its effect on the chymosin production

**Figure 4:**
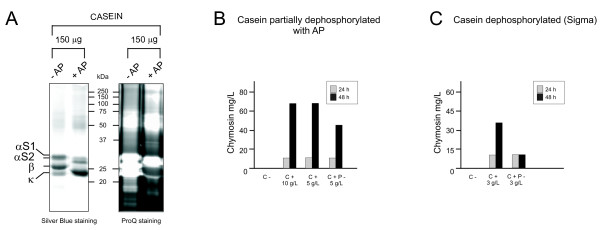
**Dephosphorylation of casein with "antartic" alkaline phosphatase and effect of the dephosphorylated casein on chymosin secretion**. **A**. SDS-PAGE (12%) of casein and dephosphorylated casein after alkaline phosphatase treatment. Four subunits of casein were detected after blue-silver Colloidal Coomassie staining (left). ProQ Diamond Staining (rigth) showed a significant decrease in the signal intensity of the bands corresponding to the dephosphorylated subunits of casein (subunits β and κ) after treatment with "antartic" alkaline phosphatase (lane +AP). Tandem mass spectrometry was used to identify the casein subunits. **B**. Production of chymosin at 24 and 48 h in cultures without any addition, supplemented with 10 g/L or 5 g/L casein (C+) or with 5 g/L partially dephosphorylated casein (C+P-). **C**. Production of chymosin at 24 and 48 h using MDFA3 medium without supplement, supplemented with 3 g/L casein, or with 3 g/L commercially dephosphorylated casein (Sigma).

Since the stimulatory effect of casein on chymosin production might be due to phosphorylated peptides, we tested whether this effect was correlated to the casein phosphorylation rate. For this purpose, dephosphorylation of casein and CPPs was performed using the "antartic" alkaline phosphatase (New England Biolabs). The dephosphorylation efficiency was assessed by running SDS-PAGE gels, which were stained with ProQ (specific dye of phosphorylated peptides) and with blue-silver Colloidal Coomassie (unspecific stain). A large-scale alkaline phosphatase treatment of casein resulted in incomplete dephosphorylation, particularly of the high-molecular weight bands, which corresponded to the αS1 and αS2 subunits (Figure 4A). On the other hand, β and κ subunits were not visible after the specific ProQ staining, suggesting full dephosphorylation of these subunits.

Cultures of the TAPL-4 strain were carried out using MDFA3 medium supplemented either with 5 g/L and 10 g/L casein or with 5 g/L partially dephosphorylated casein. Using 5 g/L and 10 g/L casein, a production of 69.1 mg/L was achieved after 48 h (Figure 4B), whereas using 5 g/L partially dephosphorylated casein, the production of chymosin decreased to 46.08 mg/L at 48 h. A second experiment was performed using commercially dephosphorylated casein (Sigma), which has a dephosphorylation degree of at least 70%. As it can be observed in Figure 4C, chymosin production at 48 h in those cultures supplemented with commercially dephosphorylated casein was clearly lower (less than 40%) than in casein-supplemented cultures. In conclusion, it seems that the stimulatory effect of casein on chymosin production is largely due to CPPs.

Phosphorylated peptides similar to CPPs occur in other proteins, although they have not been studied in detail. Bovine seroalbumin (BSA), but not soy peptones, produces a similar effect in chymosin production. BSA (fraction V Sigma) exerted the same stimulatory effect as casein in the T7-36, TAPL-4 and TG-87 strains. This stimulatory effect was lower than that provided by casein after 24 h. However, this effect increased at 48 h, yielding high chymosin productions (up to 69.1 mg/L) in the three strains (data not shown).

### Casein does not increase the transcription of the chy gene

**Figure 5:**
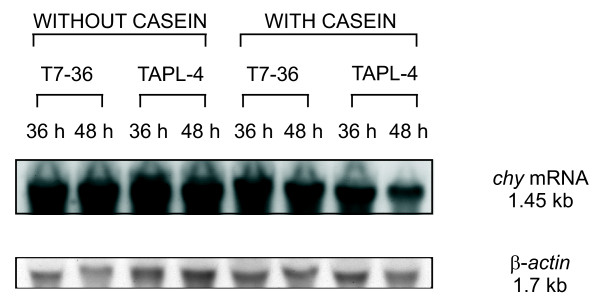
**Northern blot analysis showing the expression of the *chy *and *β-actin *(control) genes in T7-36 and TAPL-4 strains**. Total RNA was extracted at 36 and 48 h from cultures grown in MDFA3 medium either supplemented or not supplemented with 10 g/L casein. Analysis of the *chy *gene transcripts was performed using the 736-bp *Stu*I-*Kpn*I probe that is internal to the *chy *gene. For the analysis of the β-actin mRNA, the 834-bp *Nco*I-*Kpn*I fragment of the *A. nidulans β-actin *gene was used as probe. Note that similar expression levels are provided with and without casein.

In order to test whether casein induces the expression of the *chy *gene, northern blot analyses were carried out. RNA samples from T7-36 and TAPL-4 strains were taken at 36 and 48 h from cells grown in the presence and absence of casein. In these strains, the *chy *gene is expressed from different promoters. Therefore, the possibility that casein induction was exerted at the transcription level seemed to be unlikely. A 736-bp DNA fragment that is internal to the *chy *gene, was used as probe. Another probe (834-bp) contained the *A. nidulans β-actin *gene, whose expression was used as a control of the RNA loaded in each lane. Northern blot analyses revealed that no significant differences were observed in the expression of the *chy *gene (1.45-kb) either in the presence or absence of casein in the culture media (Figure 5). Similar results were obtained with CPPs (data not shown). Therefore, it can be concluded that casein or CPPs exert a positive effect on chymosin secretion, although they do not induce the expression of the *chy *gene.

### Western blot analysis shows an increased secretion of chymosin in casein-supplemented or CPPs-supplemented cultures

**Figure 6:**
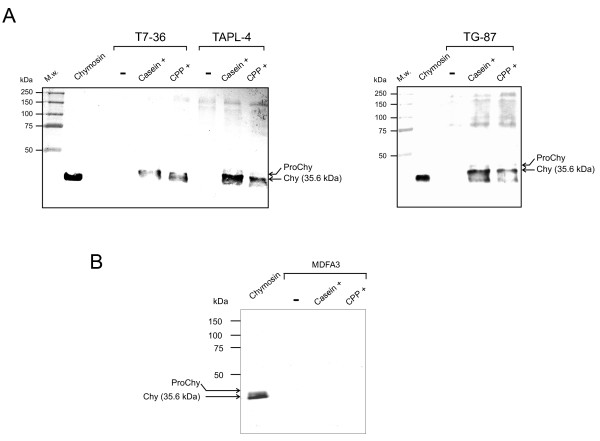
**Immunoblot detection of chymosin**. **A**. Samples were taken at 48 h from TAPL-4, T7-36 and TG-87 strains grown in MDFA3 medium without (-) or with either 5 g/L casein or 5 g/L CPPs (CPP+). Antichymosin rabbit antibodies were used. The mature protein was identical to the natural bovine chymosin (35.6 kDa). The band of unprocessed prochymosin (barely visible) is also indicated by a bent arrow. **B**. MDFA3 culture medium supplemented without (-) or with either 5 g/L casein or 5 g/L CPPs (CPP+).

If the effect of casein or CPPs is not exerted at the transcriptional level, it must be a consequence of a post-transcriptional event. To confirm this hypothesis, the culture supernatants of T7-36, TAPL-4 and TG-87 strains were analyzed by western blot using polyclonal antibodies raised against the bovine chymosin [18]. As shown in Figure 6A, those culture supernatants obtained after the addition of casein or CPPs showed an immunoreactive band with the same mobility as the authentic mature calf chymosin (35.6 kDa). No immunoreactive bands with a size coincident with that of chymosin were detected in those supernatants that were obtained from control cultures. However, faint immunoreactive bands at high apparent molecular weight are visible for some samples (particularly in strain TAPL-4). In order to ascertain whether these bands are related to casein, CPPs, chymosin or other proteins produced by the fungus, a negative control with non-inoculated casein/CPPs-containing medium was made (Figure 6B). The MDFA3 "clean" medium itself or supplemented with either casein or CPPs did not show any band after western blot analysis. This suggests that those high-molecular-weight bands may result from unspecific hybridization of the antichymosin antibodies with fungal proteins, although they most likely correspond to complexes of the unprocessed prochymosin form, as it was previously reported [18]. These results, taken together, confirm that casein or CPPs addition leads to an increase in the secretion of chymosin. This is in agreement with the increased clotting activity that was observed for the supernatants of either CPPs- or casein-supplemented cultures (Figure 2B).

### Effect of CPPs on the intracellular and extracellular proteomes of A. awamori

**Figure 7:**
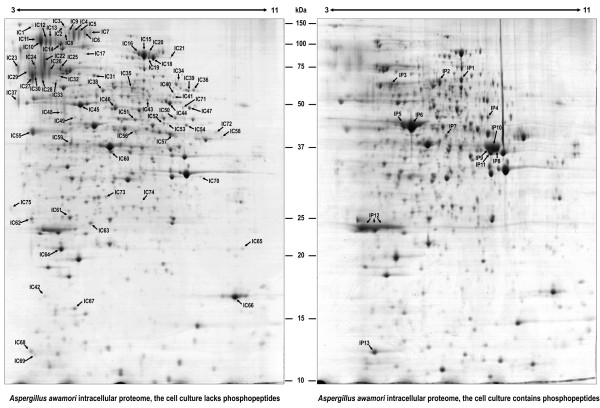
**Changes in the intracellular proteome of *A. awamori *produced by the addition of CPPs (10 g/L)**. Proteins were separated by 2-DE. The abbreviation "IC" is used for those spots underrepresented or absent in the CPPs-supplemented cultures, whereas "IP" is used for those spots overrepresented or present only after CPPs addition. The spots differentially represented at each time point are numbered and correspond to those proteins listed in Additional File 2 (Table S1 for IC and Table S2 for IP).

Proteomics studies were conducted to seed light into the mechanisms that lead to the increased chymosin secretion after phosphopeptides addition. Both intracellular and extracellular protein fractions were analyzed by 2-DE and tandem MS in cultures with and without CPPs supplementation. Analysis of the intracellular proteome (Figure 7) showed that 75 spots (including 90 proteins) were downrepresented, whereas 13 spots (including 17 proteins) were overrepresented after the addition of CPPs (Additional File 2, Table S1 and Table S2).

**Figure 8:**
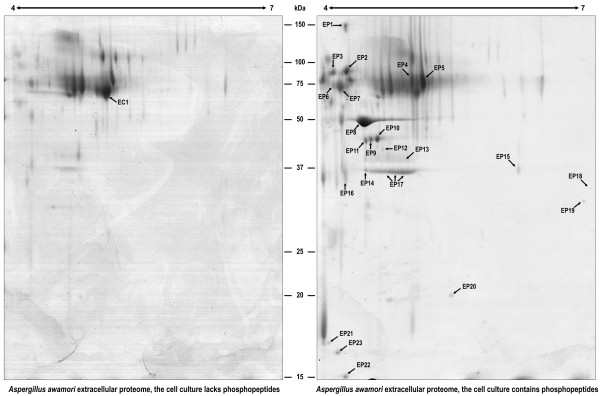
**Changes in the extracellular proteome of *A. awamori *produced by the addition of CPPs**. Proteins were separated by 2-DE. The abbreviation "EC" is used for those spots underrepresented or absent in CPPs-supplemented cultures, whereas "EP" is used for those spots overrepresented or present only after CPPs addition. The spots differentially represented at each time point are numbered and correspond to those proteins listed in Additional File 2 (Table S3 for EC and Table S4 for EP).

The analysis of the extracellular proteome (Figure 8) revealed that only one spot (including one protein) was underrepresented, whereas 25 spots (28 proteins) were overrepresented after the addition of CPPs (Additional File 2, Table S3 and Table S4). This large increase in the abundance of extracellular proteins following the addition of CPPs is very interesting (see below). Proteins were grouped according to functional categories and the main findings are summarized below.

### Intracellular proteins differentially represented after the addition of CPPs

#### Carbohydrate metabolism and energy

Most of the proteins downrepresented in the CPP-supplemented culture were involved in carbohydrate metabolism and aerobic energy pathways. Some examples are provided by a putative mitochondrial aconitate hydratase (spots IC15, IC16 and IC20, An08g10530), a probable enolase (spot IC45, An18g06250), a probable phosphoglycerate mutase (spot IC49, An01g14090), a probable citrate synthase (spot IC50, An15g01920) a probable pyruvate dehydrogenase E1 component alpha subunit (spot IC51, An07g09530), a dihydrolipoamide acetyltransferase component of pyruvate dehydrogenase (spot IC57, An07g02180) or a triose-phosphate isomerase (spot IC63). In addition, a probable UTP-glucose-1-phosphate uridylyltransferase (An12g00820), which is involved in carbohydrate interconversion, is found in the control culture in spots IC40 and IC41, but not in CPPs-supplemented cultures. Alcohol utilization (oxidation) proteins, like mannitol 2-dehydrogenase (spot IC38, An03g02430), alcohol dehydrogenase (spot IC58, An02g02060) or aldehyde dehydrogenase (spot IC43) were downrepresented in the supplemented cultures.

Interestingly, it seems that in cultures supplemented with CPPs, the metabolic fluxes were diverted to the anaerobic fermentative pathway. This is supported by the finding of a probable acetaldehyde-forming pyruvate decarboxylase (spot IP2, An02g06820) and three isoforms of the fermentative ADH1 alcohol dehydrogenase (spots IP8, IP9 and IP10), which are highly overrepresented after CPPs supplementation. This suggests that there is a limitation in the oxygen available in those cultures grown in the presence of phosphopeptides (see below), which leads to the conversion of glucose into ethanol by these enzymes. Glyceraldehyde-3-phosphate dehydrogenase (spot IP11) is another interesting protein overrepresented after phosphopeptides addition. Besides its key role in glycolysis, a number of roles have been reported for this enzyme, including modulation of the cytoskeleton, phosphotransferase/kinase activity, and the fusogenic activity involved in vesicle fusion [19,22-24]. All these activities are essential for the maintenance of normal secretory functions and indeed, it has been demonstrated that glyceraldehyde-3-phosphate dehydrogenase is required for vesicular transport from the ER to Golgi in the early secretory pathway [25]. Therefore, CPPs may play an important role in the stimulation of the protein secretion pathaway.

#### Amino acid and nitrogen metabolism

The finding that two different proteins with the same putative function are alternatively overrepresented under each condition is remarkable. This is the case of a putative aspartate transaminase, which is only present in the control cells (spot IC58, An04g06380) and in spot IP4 (An16g05570), which is 5-fold overrepresented in CPPs-supplemented cells. Whether the function of these two protein forms is exactly the same, is unknown. Several amino acid biosynthetic enzymes decreased in CPPs-supplemented cells; e.g. homoaconitate hydratase. This enzyme of the fungal lysine biosynthetic pathway is only detected in the control cells (spot IC17, An15g00350) (Figure 7). The NADP-dependent glutamate dehydrogenase (spot IC46), which converts glutamate to α-ketoglutarate and vice versa, is 1.81-fold downrepresented in the CPPs-supplemented cells. All these changes are consistent with a decrease of primary metabolism as a consequence of the detection of CPPs availability.

#### Other metabolic pathways

A probable S-adenosylmethionine synthetase (An08g02700) is only detected in control cultures (spot IC70). This enzyme catalyzes the formation of S-adenosylmethionine, which is an important methyl donor for transmethylations and is also the propylamino donor in polyamine biosynthesis. One of the pathways where S-adenosylmethionine acts as a methyl donor is the ubiquinone biosynthesis. In this pathway, a molecule of 4-hydroxybenzoate undergoes a prenylation, a decarboxylation and three hydroxylations alternating with three methylation reactions, resulting in the formation of coenzyme Q [26]. Interestingly, a putative 3-polyprenyl-4-hydroxybenzoate decarboxylase, which is involved in the biosynthesis of this coenzyme, is 10.7-fold overrepresented in the cultures supplemented with phosphopeptides.

#### Nutrient acquisition

A global overview of the proteins that are downrepresented or fully absent in the intracellular proteome of CPPs-supplemented cultures shows that *pre*-enzymes related to nutrient acquisition are more abundant in the intracellular fraction of control cells, specially several probable acid phosphatases, phosphoesterases and phytases (spots IC4-IC9, *aphA*; spots IC10-IC12, An08g0985; spot IC13, An18g04140; spots IC22, IC26 and IC28, An01g14940; spots IC29 and IC30, phyB; spots IC37 and IC42; An12g10630) (Figure 7). This suggests that when phosphopeptides are added, the intracellular concentration of these enzymes is decreased due to enhanced secretion (see below). Glucoamylase, an abundant enzyme in *A. awamori*, which hydrolyzes 1,4-alpha- and 1,6-alpha-glucosidic linkages in starch yielding glucose, is detected in the intracellular protein extracts of control cultures, but is absent in CPPs-supplemented cells (spots IC11, IC12 and IC13). The alpha-galactosidase (hydrolysing α-1,6-linked galactose residues from oligomeric compounds) is also downrepresented in intracellular extracts of CPPs-supplemented cells (spots IC22, IC24, IC29 and IC30). These results suggest that glucoamylase and galactosidase are depleted from the cytoplasm by increasing their secretion in a mechanism mediated by phosphopeptides.

Correlated to this, is the finding that the protease *pepE *(spot IC55) and one probable oligopeptidase (spot IC65, An16g08150) are also downrepresented in CPPs-treated cells.

#### Protein folding, modification and targeting

It is known that overproduction of recombinant proteins in eukaryots induces the overexpression of genes encoding several chaperones including BiP, a chaperone of the heat shock protein (HSP-70) family [12,27]. We found that several chaperones of the heat shock protein family (spots IC2 and IC3, An01g13220; spots IC25 and IC74, An07g09990; spots IC32, IC33 and IC48, An16g09260) and the cyclophilin-like peptidyl prolyl cis-trans isomerase, which accelerates protein folding (spot IC66) and a Hsp90 binding co-chaperone (spot IC62, An14g05320), are underrepresented or even absent in the CPPs-supplemented cultures. In addition, the lectin chaperone calnexin (spot IC24, An01g08420) was detected under control conditions, but absent in CPPs-supplemented cultures. Phosphopeptides reduce the levels of the above-mentioned proteins, probably reflecting a more efficient system of protein secretion that reduces the unfolded proteins and the need for chaperones.

#### Response to stimuli

Oxidative stress response proteins are more abundant in control cultures than in CPPs-supplemented ones. These include catalase-peroxidase (spots IC17, IC18 and IC19, An01g01830; spot IC39, An02g02750), a mitochondrial peroxiredoxin PRX1 (spot IC63, An04g03360) and a cytochrome C peroxidase Ccp1 (spot IC73, An04g04060). This decrease of the oxidative stress proteins correlates with the change in aerobic/fermentative metabolism (see Discussion).

#### Regulatory proteins

A probable transcriptional repressor TupA/RocA, which represses asexual development and yeast cell morphogenesis [28], is only detected under control conditions but not in CPPs-supplemented cultures (spot IC57, An15g00140) (Figure 7). Another transcriptional NmrA-like repressor (spot IC60, An15g02410) was found underrepresented in CPPs-supplemented cells. NmrA is an *Aspergillus nidulans *negative transcriptional regulator involved in modulating the activity of the transcription factor AreA [29,30], which is responsible for nitrogen metabolite regulation [31,32].

### Extracellular proteins differentially represented after the addition of CPPs

Proteins in the culture medium (the secretome) were compared with and without CPPs supplementation (Figure 8).

#### Cell wall biosynthetic proteins

After phosphopeptides addition, several proteins involved in cell wall synthesis were found overrepresented in the extracellular fraction. This is the case of 1,3-beta-glucanosyltransferases An09g00670 (spot EP3) and An10g00400 (spots EP9, EP10 and EP11). The same was observed for spot EP2, a putative 1,3-beta-glucanosyltransferase [33] that did not match any of the *A. niger *predicted proteins present in databases (although it may be present in the genome of *A. awamori*). These enzymes are well known to be secreted through vesicles that are fused to the cytoplasmic membrane. Other enzymes important for proper cell wall ultrastructure and organization that were overrepresented in the extracellular fraction after phosphopeptides addition were a GPI-anchored cell wall organization protein Ecm33 (spots EP6 and EP7, An04g01230) and a GPI-anchored cell wall beta-1,3-endoglucanase EglC (spot EP16, An03g05290).

#### Proteolytic proteins and allergens

As described above, CPPs addition drastically increases the secretion of the heterologous protein chymosin. Spot EP17 (a band that was analyzed in three different positions and corresponds to chymosin) is 6.46-fold overrepresented in CPPs-supplemented cultures compared to control cultures. Two other minoritary forms of this protein (spots EP13 and EP14) and spots EP12 (aspartic protease pepE) and EP19 (serine proteinase pepC), were only detected under CPPs-supplemented conditions.

Another interesting protein secreted after the addition of phosphopeptides is a probable allergenic cerato-platanin Asp-F13 (An02g01550), which is detected in spots EP21, EP22 and EP23. This protein was described in the ascomycete *Ceratocystis fimbriata *[34].

#### Nutrient acquisition

Two isoforms of the *alpha*-galactosidase were found overrepresented after phosphopeptides addtion (spots EP4 and EP5). This protein was underrepresented in the intracellular proteome of CPPs-supplemented cells. The *alpha*-Amylase was only detected in the extracellular proteome of CPPs-supplemented cultures (spot EP8) and represents the spot with the higher intensity, thus confirming the beneficial effect of phosphopeptides on protein secretion. Since α-amylase is an endogenous *A. awamori *protein (encoded by the duplicated *amyA *and *amyB *genes) it is concluded that the stimulatory effect of CPPs on the secretion of proteins is common for homologous and heterologous proteins.

The only extracellular protein found underrepresented in the extracellular proteome of CPPs-supplemented cells is a probable phosphoesterase (spot EC1, An01g14940). Strikingly, another isoform of the same protein was only found after phosphopeptides addition (spot EP4), suggesting that CPPs addition causes a modification of this protein.

## Discussion

CPPs are fragments of casein that are rich in phosphoserine [35,36]. They are formed by partial hydrolysis of casein by the *A. awamori *proteases. These phosphoserine residues bind calcium and indeed, CPPs increase the intestinal absorption of calcium in rats [37,38]. Further characterization of the CPPs led to the isolation of β-casein(1-25) and α-casein(59-79) (the numbers in parenthesis correspond to the amino acids in the casein subunits), two peptides rich in phosphoserine with immunomodulating activity [39-41]. Phosphoserine itself, however, was not able to increase the secretion of chymosin, indicating that the secretory pathway is induced by the phosphorylated peptides that are present in CPPs, rather than by a specific phosphorylated amino acid.

Secretion of heterologous proteins in fungi is a subject of great interest because they are usually secreted with a 10 to 100-fold lower efficiency than homologous proteins [8,13,42]. The reasons for these differences appear to be related to the low adaptation of the heterologous proteins to the characteristics of the fungal secretory pathway. Endogenous proteins seem to be better adapted. For this study, we have used the secretion of bovine chymosin as a model. Surprisingly, we found that CPPs (or casein) drastically stimulate (from 40 to 80-fold depending on the strains and culture conditions) the production of extracellular chymosin. This effect was not exerted by casamino acids (fully hydrolyzed casein) or by the ammonium salts used as nitrogen source for growth.

Northern analysis of the expression of the *chy *gene from two different promoters (*gpdA *or *amyB*) revealed that CPPs do not significantly change the transcription of the *chy *gene. However, western blot analysis confirmed the increased secretion of chymosin (prochymosin and mature chymosin forms) following the supplementation with CPPs.

It is well known that CPPs elicit the secretion of lymphokines in cultures of intestine epithelial cells [21]. It has also been reported that CPPs inhibit gastric secretion probably by reaching target sites (e.g. receptors, enzymes) at the luminal site of the gastrointestinal tract [43]. However, the mechanisms involved are not known. Therefore, we have investigated the proteomics of the cellular response to CPPs addition. The proteomics studies revealed major changes in the *A. awamori *metabolism, particularly in the depletion of pre-enzymes in the intracellular proteome and large increases in the representation of many extracellular proteins in the secretome.

One of the primary changes observed, was the cellular response to oxygen. Several oxidative stress response proteins (catalase and peroxidase) are underrepresented in the proteome of CPPs-supplemented cells. Oxidative stress and the occurrence of reactive oxygen species are common to aerobically living organisms. However, during fermentation studies we also showed that the dissolved O_2 _levels present in the culture medium were higher after CPPs addition, which indicates that there is a limitation in the use of oxygen by the cell, probably due to changes in membrane permeability. Proteomics studies also suggested that in cultures supplemented with CPPs, the metabolic fluxes were diverted to the anaerobic fermentative pathway. This is supported by the overrepresentation in CPPs-supplemented cultures of a protein similar to the bacterial hemoglobin (spots IP5 and IP6). A major problem in industrial fermentations with *A. awamori *is to ensure sufficient oxygen supply, which is required for respiratory metabolism. This is the reason why in the case of oxygen limitation, the fungus will produce various by-products, such as reduced organic acids and alcohols. It has been reported that the overexpression of the gene encoding a bacterial hemoglobin from *Vitreoscilla *in *Acremonium chrysogenum *or in *A. niger *leads to a relief of stress when the transformants were exposed to oxygen limitation [44,45]. These hemoglobin proteins enhanced the production of alpha-amylase by the yeast *Schwanniomyces occidentalis *[46]. CPPs may produce a similar effect by increasing the concentration of the endogenous *A. awamori *hemoglobin-like protein.

There is also a significant change in sugar metabolism. We found in CPPs-supplemented cells a decrease in several enzymes related to glycolysis and citric acid cycle (phosphoglycerate mutase, citrate synthase, mitochondrial aconitate hydratase, among others), whereas there is an increase in several enzymes that catalyze the conversion of pyruvic acid to ethanol (e.g. pyruvate decarboxylase [forming acetaldehyde] and three isoforms of the alcohol dehydrogenase).

A noteworthy change is that of the glyceraldehyde-3-phosphate dehydrogenase, which is overpresented in the proteome of CPPs-supplemented cells. This enzyme has been reported to play several roles in metabolism including a vesicle fusogenic activity and a cytoskeleton-modulation phosphotransferase/kinase activity [19,22-24]. Therefore, it has been included in the group of "moonlighting proteins", a designation for the collective of proteins with two or more unrelated functions that are widespread among organisms ranging from bacteria to mammals [47,48]. This primarily intracellular protein has been reported to be secreted by a non-conventional system in the fungus *Penicillium chrysogenum *[49]. The exact mechanism of glyceraldehyde-3-phosphate dehydrogenase that is related with cytoskeleton extension and vesicle traffic is still unknown. However, a role of this protein in the secretory protein traffic through vesicles is likely, probably by facilitating vesicle targeting to appropriate cell membrane locations and fusion to the cell membrane.

Our results show a very clear effect of CPPs on depletion of pre-enzymes from the intracellular space and on their release to the extracellular medium. Several proteins involved in cell-wall biosynthesis that are targeted to and released at the tip of the hyphae, are overrepresented in CPPs-supplemented cells. In *Neurospora crassa*, vesicles aimed for fusion at the growing hyphal tip show high concentration near the apex (0 to 5 μm). However, they exponentially decline at increasing distances from the tip [50]. An organellar Ca^2+ ^gradient concentration, which is required for hyphal tip growth, occurs in the hyphal tip region [51,52]. CPPs are known to form complexes with Ca^2+ ^and this may be the molecular mechanism of their action on vesicle-mediated secretion of cell-wall related enzymes.

Another large group of extracellular proteins overrepresented in CPPs-supplemented cultures include α-amylase and extracellular proteases. A member of this group is the heterologous chymosin that shows several isoforms with the same molecular mass but with different charge in the secretome 2D gels. Two minoritary secreted chymosin forms with slightly larger molecular mass correspond to pro-chymosin forms. It is noteworthy that the intracellular pre-enzyme forms of several extracellular enzymes detected in the proteome of control cells are absent or greatly reduced in the intracellular proteome of CPPs-supplemented cells, confirming the enhanced secretion of these proteins. The secretion of α-amylase (the majoritary protein in extracellular extracts), is also enhanced in the CPPs-supplemented cultures, indicating that the stimulation of the secretion is a general mechanism that affects both minoritary and abundant proteins.

## Conclusions

Our results indicate that CPPs trigger a signaling cascade that changes the carbohydrate metabolism, oxygen utilization and the activity of the secretory pathway. It is noteworthy that some overrepresented proteins in the secretome of CPPs-supplemented cultures are extracellular enzymes, which are targeted to the hyphal tips. On this location, they fuse to the cell membrane releasing the active enzymes. This work provides a background for important improvements of valuable heterologous protein secretion in filamentous fungi.

## Methods

### Microorganisms and culture conditions

*A. awamori lpr*66, a strain deficient in aspergillopepsin A [42] was used as the host strain for transformation with expression vectors containing the *chy *gen. *E. coli *DH5α was used for plasmid amplification.

*A. awamori *transformants were grown on solid medium for sporulation as previously described [18]. The defined MDFA3 chymosin production medium contained: 10 g/L glucose; 20 g/L starch; 10,57 g/L ammonium sulfate; 144 mL salts II (104 g/L K_2_HPO_4_; 102 g/L KH_2_PO_4_; 5,08 g/L Na_2_SO_4_) and 144 mL salts III (2,4 g/L MgSO_4_.7H_2_O; 0,2 g/L ZnSO_4_.7H_2_O; 0,2 g/L MnSO_4_.7H_2_O; 0,05 g/L CuSO_4_.5H_2_O and 0,37 g/L CaCl_2_). This medium was inoculated with 9% of seed culture [18] and cultures were grown at 30°C and 250 rpm for 48 h in a rotary shaker (Model 481 Console incubator/refrigerated shaker, Thermo Scientific). MDFA3 was supplemented with different compounds as explained in the Results section.

For *A. awamori *fermentations, seed cultures (9%) were used to inoculate three 5-L Biostat B (Braun, Germany) fermentors, which contained 3 L of defined MDFA3 medium without or with the presence of either 10 g/L casein or 10 g/L CPPs. The fermentations were run at 30°C and 300 rpm with an air flow of 2,5 L/min. The agitation speed was increased to 350 rpm at 24 h and 500 rpm at 36 h.

### Plasmid construction for chy gene expression and transformation of A. awamori

For the construction of pGlaQF, a 1146-bp fragment corresponding to the coding region of the preprochymosin, was synthesized by PCR using primers PreQ1 (5'CGC TGC CTG GTC CTG CTG GCC3') and TQ3 (5'TCA AAT GGC CTT GGC CAG ACC GAC3'). Plasmid pANPPQ carrying the complete cassette of the *chy *gene under the *gpdA *promoter, was used as template [18]. Next, DNA was digested with *Kpn*I, giving rise to a 450-bp fragment that was ligated to plasmid pJL43b7 (kindly provided to us by S. Gutiérrez, University of León, Spain), which contains the *A. niger gla *gene promoter fused to the *ble *gene (for phleomycin resistance) and the *S. cerevisiae cyc1 *transcriptional terminator. This plasmid was previously digested with *Nco*I, blunt-ended with Klenow and digested with *Kpn*I. The resultant plasmid, pJL43bq, carries the *glaA *promoter fused to the pre-prochymosin sequences and part of the *chy *gene. Then, p84AB (kindly provided to us by R.E. Cardoza, University of León, Spain), was digested with *Kpn*I, giving rise to a fragment of 1045 bp containing the last 700 bp of the *chy *gene fused to the *S. cerevisiae cyc1 *gene transcriptional terminator. This fragment was ligated to the *Kpn*I site of pJL43bq to obtain pGlaQ, which bears the complete chymosin expression cassette under the control of the *glaA *promoter. pGlaQ was digested with *Bam*HI and *Hin*dIII and the chymosin expression cassette was inserted into pJL43b1 (containing a phleomycin resistance cassette: PgpdA::ble::Tcyc) [53], wihch was digested with the same enzymes resulting in the final plasmid pGlaQF.

The *amyB *promoter (800-bp) was obtained from plasmid pTAex3 [54] after *Bam*HI digestion. Then, it was subcloned into pBluescript SK+ (digested with the same enzyme), thus giving rise to plasmid pTA16. Simultaneously, a 1146-bp PCR fragment containing the preprochymosin, was generated using two oligonucleotides; ProQ1 (5'GCC GAG ATC ACC CGC ATC CCC CTG3') and TQ3 (5'TCA AAT GGC CTT GGC CAG ACC GAC3'). Plasmid pANPPQ was used as template. After digestion with *Cla*I, a 917-bp fragment was introduced into the *Sma*I-*Cla*I-digested pTA16, obtaining plasmid pTAmy, which carries the *amy*B gene promoter fused to pre-pro sequences of the *chy *gene (it also includes a fragment of the *chy *gene). The final part of the *chy *gene and T*cyc*1 were excised from *Cla*I-digested pGlaQ, giving rise to a 538 bp fragment, which was ligated to the *Cla*I-digested pTAmy to obtain pAQ. The complete expression cassette of the *chy *gene under the control of *amy*B promoter was then ligated as a *Bam*HI-*Hin*dIII fragment to the phleomycin resistance cassette, which was obtained from pGlaQF (excised with the same enzymes), thus giving rise to plasmid pAQF.

To obtain plasmid pGlaPLQF, PCR was first performed using plasmid pThIX as template [55]. It contains the *glaA *gene promoter and its leader peptide. Primers PGLA (5'TTT GGA TCC GAA CTC CAA TCG GGG GGA3') and phopshorylated PLGLA2-P (5'TGC CAA CCC TGT GCA GAC GAG GCC3') were also used. The 854-bp product carrying the *glaA *gene promoter and its leader peptide was digested with *Bam*HI. The coding region of the prochymosin was obtained by PCR using pANPPQ as template and primers PROQ2-P (5'GCC GAG ATC ACC CGC ATC CCC CTG3') and TQ3 (see above). The 1098-bp amplified product was digested with *Cla*I to obtain the fragment carrying the coding region of the pro sequence of the *chy *gene (it also includes a fragment of the *chy *gene). These two PCR products that were previously digested, were ligated to the pGlaQ vector, which was digested with *Bam*HI and *Cla*I to give rise to pGlaPLQ. Next, pGlaPLQ was digested with *Bam*HI and *Hin*dIII. This fragment was inserted into pGlaQF (containing a phleomycin resistance cassette) previously digested with the same enzymes, to obtain pGlaPLQF.

For the construction of pAPLQF, the region including the *A. oryzae amyB *gene promoter and its leader peptide, was amplified with primers PAMY2 (5'TTT TGG ATC CCC ATC ATG GTG TTT TGA T3') and phopshorylated PLAP (5'AGC CAA AGC AGG TGC CGC GAC CTG3'). Genomic DNA from *A. oryzae *was used as template. The 680-bp fragment amplified by PCR was digested with *Bam*HI. The coding region of the prochymosin was obtained by PCR using pANPPQ as template and primers PROQ2-P and TQ3 (see above). This fragment was also cut with *Cla*I. These two PCR products previoiusly digested, were ligated to pGlaQ, digested with *Bam*HI and *Cla*I to generate pAPLQ. Finally, pGlaQF was digested with *Bam*HI and *Hin*dIII and ligated to the *Bam*HI and *Hin*dIII fragment of pAPLQ, which corresponds to the complete chymosin expression cassette with the *amyB *gene promoter and its leader peptide, thus giving rise to plasmid pAPLQF.

Plasmids were introduced into *A. awamori *by transformation as described by Cardoza and co-workers [18].

### RNA Isolation and Northern Hybridizations

Total RNA was isolated as described by Chomczynski and Sacchi [56] from frozen mycelium that was disrupted by grinding it in a mortar previously refrigerated with liquid nitrogen. The disrupted cells (0.5 to 1 g) were mixed with 1.3 ml of the EFA solution containing equal volumes of phenol and extraction buffer (4 M guanidinium thiocyanate, 0.5% (w/v) lauryl sarcosine and 25 mM sodium citrate, pH 7.0). Seven μl of β-mercaptoethanol were added for each mL of extraction buffer. The mixture was centrifuged at top speed in a microcentrifuge for 5 min and the supernatant was mixed with 130 μl chloroform-isoamyl alcohol (CIA) (24:l, vol/vol). The mixture was centrifuged again and the supernatant was purified by phenol-CIA and CIA extractions. One-half volume of 8 M LiCl was added to the clarified supernatant and precipitated overnight at 4°C. RNA was recovered by centrifugation, washed with 70% (v/v) ethanol and resuspended in Milli-Q water. Northern hybridizations were performed according to Sambrook and Russell [57]. Probes were internal to the synthetic *chy *gene (*Stu*I-*Kpn*I fragment) and to the *β-actin *gene of *A. nidulans *(*Nco*I-*Kpn*I fragment).

### SDS-Electrophoresis and western blotting

SDS-Electrophoresis and western blotting were carried out as previously described [18]. For phosphoprotein detection, SDS-PAGE gels were stained with Pro-Q Diamond gel dye according to the modified protocol of Eymann and co-workers [58]. Gels were scanned using the FCA 5000 FujiFilm reader (Excitation source: 532 nm laser, longpass emission filter: 555 nm) with a resolution of 50 micron.

### Milk-Clotting Assay and Chymosin Quantification

Chymosin was quantified by milk clotting. Serial dilutions (1/2) of a pure preparation of chymosin (500 ng) were prepared in a 96-well microtiter plate. Each well contained 175 μl. The number of wells where milk clotting takes place (white precipitate) is proportional to the concentration of chymosin. The minimal dilution that produces milk clotting in the assay is the fourth chymosin dilution (1/16), which corresponds to 31.25 ng chymosin (500/16 = 31.25). This amount of chymosin corresponds to a concentration of 0.18 mg/L (31.25 ng chymosin/175 μl). Therefore, for the quantification of the chymosin produced by different strains of *A. awamori*, serial dilutions (1/2) of the culture broths were prepared. Then, 0.18 was multiplied by the corresponding dilution that produces clotting in each assay. Due to the steps in sample dilution, estimation of chymosin production cannot be strictly exact and therefore, production was expressed as the range of concentrations corresponding to two consecutive dilutions (i.e.: if the last dilution of culture supernatants that provides clotting activity is 1/256, the estimated concentration of chymosin would range from 46.08 mg/L [0.18 × 256] to 92.16 mg/L [0.18 × 512]). We always consider the lowest amount (i.e. 46.08) as the coagulation-positive activity because the following dilution step does not give rise to clotting reaction.

### Protein extraction protocol for proteomics studies- A. awamori

TAPL-4 (see Results) was grown as previously described [18]. Briefly, this strain was grown for sporulation at 30°C for 3 days on Power solid medium, which was obtained by mixing 1:1 (v/v) standard Czapek medium and PM1 medium (30 g lactose, 5 g Bacto Peptone, 0.5 g corn steep solids, 4 g NaC1, l mg CuSO_4_·7H_2_0, 3 mg FeC1_3_·6H_2_0, 60 mg KH_2_PO_4_, 50 mg MgSO_4_·7H_2_0, 30 g agar, 1 L distilled water, pH 6.5). A total of 10^7 ^spores/mL were cultured in PMMY medium [59] either in the absence or in the presence of 10 g/L CPPs, and grown at 30°C and 250 rpm in a rotary shaker (Model 481 Console incubator/refrigerated shaker, Thermo Scientific). Mycelia were harvested after 48 h of growth by centrifugation. Pellets were stored (-80°C) to analyze the intracellular proteome, whereas supernatants were processed to study the extracellular proteome as follows: Supernatants were passed through a Nytal cloth filter and immediately centrifuged at 4°C and 4500 rpm for 5 min to remove any remaining mycelia. Then, they were refrigerated on ice and filtered through 0.45 μm membrane filters (Millipore). An equal volume of pre-chilled 20% (w/v) trichloroacetic acid in acetone containing 0.14% (w/v) DTT was added to the filtered supernatants, mixed and incubated overnight at -20°C. Proteins were collected by centrifugation at 4500 rpm for 5 min and 4°C. The pellet was washed twice with pre-chilled acetone containing 0.07% (w/v) DTT and once with 80% (v/v) acetone/Milli-Q-water.

Intracellular proteins were obtained following the protocol developed by Jami and co-workers [59]. After grinding the frozen mycelia to a fine powder in a pre-cooled mortar using liquid nitrogen, two grams of the final powder was resuspended (stirred at 4°C for 2 h) in 10 ml of 10 mM potassium phosphate buffer (K_2_HPO_4_**:**KH_2_PO_4_; pH 7.4) containing 0.07% (w/v) DTT and supplemented with one tablet of the protease inhibitor mixture COMPLETE™ (Roche AppliedScience). The mixture was clarified twice by centrifugation at 13200 rpm for 5 min. Extracellular proteins were obtained from the same cultures following the protocol of Jami and co-workers [49]. After nytal filtration, supernatans were centrifuged twice (3900 rpm for 5 min and 13200 rpm for 5 min) at 4°C and filtered through 0.45 μm filters. Intracellular and extracellular proteins were precipitated, washed and resuspended in sample buffer as described previously [49,59]. Samples were stored at -80°C. Protein concentration was estimated according to the Bradford method.

### 2-DE gel electrophoresis and analysis of differential protein expression

Bidimensional electrophoresis and protein determination were made as described by Jami and co-workers [59] by loading 450 μg of soluble proteins in the sample buffer (see above) onto 18-cm IPG strips (GE Healthcare) with non linear (NL) pH 4-7 gradient (extracellular proteome) or NL pH 3-10 (cytoplasmic proteome). The second dimension SDS-PAGE was run in 12.5% polyacrylamide in an Ettan Dalt Six apparatus (GE Healthcare). Gels were dyed with Colloidal Coomassie (CC) [60], which provides high reproducibility.

2-DE gels were scanned using an ImageScanner II (GE Healthcare), digitalized with Labscan 5.00 (v1.0.8) software (GE Healthcare) and analyzed with the ImageMaster™ 2D Platinum v5.0 software (GE Healthcare). Three gels of each condition obtained from three independent cultures (biological replicates) were analyzed to guarantee representative results. After automated spot detection and manual revision, relative volumes were used to quantify and compare the gel spots. Proteins were considered to be differentially expressed when the ratio of the relative volume average (three gels) between strains was higher than 1.5 and the p-value was < 0.05.

### Protein Identification by peptide mass fingerprint and tandem MS

Protein spots of interest were manually excised from the Colloidal Commassie-stained gels, digested following the method of Havlis and co-workers [61] and processed for further analysis as indicated before [59]. Samples were analyzed with a 4800 Proteomics Analyzer MALDI-TOF/TOF mass spectrometer (Applied Biosystems). The produced peptide mass fingerprints were collected and represented as a list of monoisotopic molecular weights using the 4000 Series Explorer v3.5.3 software (Applied Biosystems). Hence, from each MS spectra, the ten most intensive precursors were selected for MS/MS analyses with CID (atmospheric gas was used) in 2-kV ion reflector mode and precursor mass windows of ± 7 Da. For protein identification, Mascot Generic Files combining MS and MS/MS spectra were automatically created and used to interrogate a non-redundant protein database using a local license of Mascot v 2.2 from Matrix Science through the Protein Global Server (GPS) v 3.6 (Applied Biosystems). Search parameters for the peptide mass fingerprints and tandem MS spectra obtained were set as follows: (i) NCBInr (2009.11.03) sequence databases were used; (ii) taxonomy: All entries (9993394 sequences, 3409286210 residues); (iii) fixed and variable modifications were considered (Cys as S carbamidomethyl derivative and Met as oxidized methionine); (iv) one missed cleavage site was allowed; (v) precursor tolerance was 100 ppm and MS/MS fragment tolerance was 0.3 Da; (vi) peptide charge: 1+; and (vii) the algorithm was set to use trypsin as the enzyme. When the global Mascot score was greater than 83 with a significance level of p < 0.05 protein candidates were considered valid. Additional criteria for confident identification were that the protein match should have at least 15% sequence coverage; for lower coverage, only those proteins with a Mascot ions score above 54 and at least two peptides identified in the tandem MS analysis (with a significance level of p < 0.05), were considered valid.

## List of abbreviations

Ble: Phleomycin; BSA: Bovine seroalbumin; CC: Colloidal Coomassie; Chy: Chymosin; CIA: chloroform-isoamyl alcohol; CPPs: Casein phosphopeptides; DTT: 1,4-dithio-D-threitol; ER: Endoplasmic reticulum; Gla: Glucoamylase; MALDI-TOF: Matrix-assisted laser desorption/ionization-Time of Flight

## Competing interests

The authors declare that they have no competing interests.

## Authors' contributions

KK performed main experiments, carried out the data analysis and wrote the initial part of the manuscript. CGE was involved in data analysis and interpretation, wrote the results of the proteomic studies and revised the manuscript. CB carried out in-gel digestions and mass-spectrometric analyses. MGF participated in plasmid construction and transformants selection. MSJ carried out the proteome sample preparation and the 2-DE analyses. MAP contributed to dephosphorylation experiments. JFM designed and conducted this research project, being also responsible for manuscript preparation, writing and revision. All authors read and approved the final manuscript.

## Supplementary Material

Additional file 1 **Additional Figure 1. Milk clotting assay for casein, casamino acids, NZ Amino A or phosphoserine**. **A**. Serial dilutions (1/2) of a pure preparation of chymosin (500 ng) and the corresponding concentration in mg/L. **B**. Effect of casein, casamino acids, NZ Amino A and phosphoserine on chymosin production by strains TG-87 and TAPL-4. Culture supernatants from these strains were serially diluted from 1/2 to 1/512 (DF). No dilution is denoted as WD. The number of wells where milk clotting takes place (white precipitate) is proportional to the concentration of chymosin that is present in the culture supernatant. The chymosin concentration range that is present in those wells with clotting, has been estimated using the data obtained from panel A as it is indicated in the Methods section. Click here for file

Additional file 2 **Supplementary tables, Table S1**. Intracellular proteins overrepresented in the absence of phosphopeptides. Fold change and p-value are indicated for those proteins present in both strains. Otherwise, non-available (N/A) is shown. **Table S2**. Intracellular proteins overrepresented in the presence of phosphopeptides. Fold change and p-value are indicated for those proteins present in both strains. Otherwise, non-available (N/A) is shown. **Table S3**. Extracellular proteins overrepresented in the absence of phosphopeptides. Fold change and p-value are indicated for those proteins present in both strains. Otherwise, non-available (N/A) is shown. **Table S4**. Extracellular proteins overrepresented in the presence of phosphopeptides. Fold change and p-value are indicated for those proteins present in both strains. Otherwise, non-available (N/A) is shown. Click here for file

## References

[B1] Machida M Asai K Sano M Tanaka T Kumagai T Terai G Kusumoto K Arima T Akita O Kashiwagi Y Abe K Gomi K Horiuchi H Kitamoto K Kobayashi T Takeuchi M Denning DW Galagan JE Nierman WC Yu J Archer DB Bennett JW Bhatnagar D Cleveland TE Fedorova ND Gotoh O Horikawa H Hosoyama A Ichinomiya M Igarashi R Genome sequencing and analysis of *Aspergillus oryzae* Nature 2005 438 1157 1161 10.1038/nature04300 16372010

[B2] Pel HJ de Winde JH Archer DB Dyer PS Hofmann G Schaap PJ Turner G de Vries RP Albang R Albermann K Andersen MR Bendtsen JD Benen JAE van den Berg M Breestraat S Caddick MX Contreras R Cornell M Coutinho PM Danchin EGJ Debets AJM Dekker P van Dijck PWM van Dijk A Dijkhuizen L Driessen AJM d'Enfert C Geysens S Goosen C Groot GS Genome sequencing and analysis of the versatile cell factory *Aspergillus niger* Nature 2007 25 221 231 10.1038/nbt1282 17259976

[B3] Archer DB Jeenes DJ Mackenzie DA Strategies for improving heterologous protein production from filamentous fungi Antonie van Leeuwenhoek 1994 65 245 250 10.1007/BF00871952 7847891

[B4] Gouka RJ Punt PJ van den Hondel CA Efficient production of secreted proteins by *Aspergillus*: Progress, limitations and prospects Appl Microbiol Biotechnol 1997 47 1 11 10.1007/s002530050880 9035405

[B5] van den Hombergh JP van de Vondervoort PJ Fraissinet-Tachet L Visser J *Aspergillus *as a host heterologous protein production: the problem of proteases Trends Biotechnol 1997 15 256 263 10.1016/S0167-7799(97)01020-2 9237405

[B6] Conesa A Punt PJ van Luijk N van den Hondel CA The secretion pathway in filamentous fungi: a biotechnological view Fungal Genet Biol 2001 33 155 171 10.1006/fgbi.2001.1276 11495573

[B7] Peberdy JF Protein secretion in filamentous fungi-trying to understand a higly productive black box Trend Biotechnol 1994 12 50 57 10.1016/0167-7799(94)90100-7 7764536

[B8] Moralejo FJ Cardoza RE Gutiérrez S Martín JF Thaumatin production in *Aspergillus awamori *by use of expression cassettes with strong fungal promoters and high gene dosage Appl Environ Microbiol 1999 65 1168 1174 10049878 10.1128/aem.65.3.1168-1174.1999 PMC91159

[B9] van den Brink HJ Petersen SG Rahbek-Nielsen H Hellmuth K Harboe M Increased production of chymosin by glycosylation J Biotechnol 2006 125 304 310 10.1016/j.jbiotec.2006.02.024 16621086

[B10] Wösten HA Moukha SM Sietsma JH Wessels JG Localization of growth and secretion of proteins in *Aspergillus niger* J Gen Microbiol 1991 137 2017 2023 1955876 10.1099/00221287-137-8-2017

[B11] Fukuda K Yamada K Deoka K Yamashita S Ohta A Horinouchi H Class III chitin synthase ChsB of *Aspergillus nidulans *localizes at the sites of polarized cell wall synthesis and is required for conidial development Eukaryot Cell 2009 8 945 956 10.1128/EC.00326-08 19411617 PMC2708462

[B12] Lombraña M Moralejo FJ Pinto R Martín JF Modulation of *Aspergillus awamori *thaumatin secretion by modification of *bipA *gene expression Appl Environ Microbiol 2004 70 5145 5152 10.1128/AEM.70.9.5145-5152.2004 15345393 PMC520887

[B13] Archer DB Peberdy JF The molecular biology of secreted enzyme production by fungi Crit Rev Biotechnol 1997 17 273 306 10.3109/07388559709146616 9397531

[B14] Ward PP Lo J-Y Duke M May GS Headon DR Conneely OM Production of biologically active recombinant human lactoferrin in *Aspergillus oryzae* Nat Biotechnol 1992 10 784 189 10.1038/nbt0792-784 1368268

[B15] Broekhuijsen MP Mattern IE Contreras R Kinghorn JR van den Hondel CAMJJ Secretion of heterologous protein by *Aspergillus niger*: production of active human interleukin-hIL6 fusion protein J Biotechnol 1993 31 135 145 10.1016/0168-1656(93)90156-H 7764298

[B16] Cullen D Gray GL Wilson LJ Hayenga KJ Lamsa MH Rey MW Norton S Berka RM Controlled expression and secretion of bovine chymosin in *Aspergillus nidulans* Bio/Technology 1987 5 369 376 10.1038/nbt0487-369

[B17] Tsuchiya K Gomi K Kitamoto K Kumagai C Tamura G Secretion of calf chymosin from the filamentous fungus *Aspergillus oryzae* Appl Microbiol Biotechnol 1993 40 327 332 7764387 10.1007/BF00170388

[B18] Cardoza RE Gutiérrez S Ortega N Colina A Casqueiro J Martín JF Expression of a synthetic copy of the bovine chymosin gene in *Aspergillus awamori *from constitutive and pH-regulated promoters and secretion using two different pre-pro sequences Biotechnol and Bioengineer 2003 83 249 259 10.1002/bit.10666 12783481

[B19] Sirover MA New insights into an old protein: the functional diversity of mammalian glyceraldehyde-3-phosphate dehydrogenase Biochim Biophys Acta 1999 1432 159 184 10.1016/S0167-4838(99)00119-3 10407139

[B20] Kawahara T Otani H Stimulatory effects of casein phosphopeptide (CPP-III) on mRNA expression of cytokines in Caco-2 cells Biosci Biotechnol Biochem 2004 68 1779 1781 10.1271/bbb.68.1779 15322363

[B21] Kitts DD Nakamura S Calcium-enriched casein phosphopeptide stimulates release of IL-6 cytokine in human epithelial intestinal cell line J Dairy Res 2006 73 44 48 16433960 10.1017/S0022029905001330

[B22] Kumagai H Sakai H A porcine brain protein (35 K protein) which bundles microtubules and its porcine brain protein as glyceraldehyde 3-phosphate dehydrogenase J Biochem (Tokyo) 1993 93 1259 1269 10.1093/oxfordjournals.jbchem.a134260 6885722

[B23] Kawamoto R Caswell A Autophosphorylation of glyceraldehydephosphate dehydrogenase and phosphorylation of proteins from skeletal muscle microsomes Biochemistry 1986 25 657 661 3955021 10.1021/bi00351a022

[B24] Hessler RJ Blackwood RA Brock TG Francis JW Harsh DM Smolen JE Identification of glyceraldehyde-3-phosphate dehydrogenase as a Ca2+-dependent fusogen in human neutrophil cytosol J Leukocyte Biol 1998 63 331 336 9500520 10.1002/jlb.63.3.331

[B25] Tisdale EJ Glyceraldehyde-3-phosphate dehydrogenase is required for vesicular transport in the early secretory pathway J Biol Chem 2001 276 2480 2486 10.1074/jbc.M007567200 11035021

[B26] Meganathan R Ubiquinone biosynthesis in microorganisms FEMS Microbiol Lett 2001 203 131 139 10.1111/j.1574-6968.2001.tb10831.x 11583838

[B27] Munro S Pelham HR An Hsp70-like protein in the ER: identity with the 78 kd glucose-regulated protein and immunoglobulin heavy chain binding protein Cell 1986 46 291 300 10.1016/0092-8674(86)90746-4 3087629

[B28] Todd RB Greenhalgh JR Hynes MJ Andrianopoulos A TupA, the *Penicillium marneffei *Tup1p homologue, represses both yeast and spore development Mol Microbiol 2003 48 85 94 10.1046/j.1365-2958.2003.03426.x 12657047

[B29] Andrianopoulos A Kourambas S Sharp JA Davis MA Hynes MJ Characterization of the *Aspergillus nidulans nmrA *gene involved in nitrogen metabolite repression J Bacteriol 1998 180 1973 1977 9537404 10.1128/jb.180.7.1973-1977.1998 PMC107119

[B30] Lamb HK Ren J Park A Johnson C Leslie K Cocklin S Thompson P Mee C Cooper A Stammers DK Hawkins AR Modulation of the ligand binding properties of the transcription repressor NmrA by GATA-containing DNA and site-directed mutagenesis Protein Sci 2004 13 3127 3138 15537757 10.1110/ps.04958904 PMC2287298

[B31] Kudla B Caddick MX Langdon T Martinez-Rossi NM Bennett CF Sibley S Davies RW Arst HN The regulatory gene *areA *mediating nitrogen metabolite repression in *Aspergillus nidulans*. Mutations affecting specificity of gene activation alter a loop residue of a putative zinc finger EMBO J 1990 9 1355 1364 1970293 10.1002/j.1460-2075.1990.tb08250.x PMC551819

[B32] Wilson RA Arst HN Mutational analysis of AreA, a transcriptional activator mediating nitrogen metabolite repression in *Aspergillus nidulans *and a member of the 'streetwise' GATA family of transcription factors Microbiol Mol Biol Rev 1998 62 586 596 9729601 10.1128/mmbr.62.3.586-596.1998 PMC98926

[B33] Coen ML Lerner CG Capobianco JO Goldman RC Synthesis of yeast cell wall glucan and evidence for glucan metabolism in a *Saccharomyces cerevisiae *whole cell system Microbiology 1994 140 2229 2237 10.1099/13500872-140-9-2229 7952174

[B34] Pazzagli L Pantera B Carresi L Zoppi C Pertinhez TA Spisni A Tegli S Scala A Cappugi G Cerato-platanin, the first member of a new fungal protein family: cloning, expression, and characterization Cell Biochem Biophys 2006 44 512 521 10.1385/CBB:44:3:512 16679539

[B35] Britton JR Kastin AJ Biologically active polypeptides in milk Am J Med Sci 1991 301 124 132 10.1097/00000441-199102000-00007 2012102

[B36] Schlimme E Meisel H Bioactive peptides derived from milk proteins. Structural, physiological and analytical aspects Nahrung 1995 39 1 20 10.1002/food.19950390102 7898574

[B37] Lee YS Noguchi T Naito H Intestinal absorption of calcium in rats given diets containing casein or amino acid mixture: the role of casein phosphopeptides Br J Nutr 1983 49 67 76 10.1079/BJN19830012 6821691

[B38] Mikkänen HM Wasserman RH Enhanced absorption of calcium by casein phosphopeptides in rachitic and normal chicks J Nutr 1980 110 2141 2148 7431116 10.1093/jn/110.11.2141

[B39] Hata I Higashiyama S Otani H Identification of a phosphopeptide in bovine αs1-casein digest as a factor influencing proliferation and immunoglobulin production in lymphocyte cultures J Dairy Res 1998 65 569 578 10.1017/S0022029998003136 9839212

[B40] Hirayama M Toyota K Yamaguchi G Hidaka H Naito H HPLC analysis of commercial casein phosphopeptides (CPP) Biosci Biotechnol Biochem 1992 56 1126 1127 10.1271/bbb.56.1126 27286389

[B41] Otani H Watanabe T Tashiro Y Effects of bovine β-casein (1-28) and its chemically synthesized partial fragments on proliferative responses and immunoglobulin production in mouse spleen cell cultures Biosci Biotechnol Biochem 2001 65 2489 2495 10.1271/bbb.65.2489 11791723

[B42] Moralejo FJ Cardoza RE Gutiérrez S Sisniega H Faus I Martín JF Overexpression and lack of degradation of thaumatin in an aspergillopepsin A-defective mutant of *Aspergillus awamori *containing an insertion in the *pepA *gene Appl Microbiol Biotechnol 2000 54 772 777 10.1007/s002530000463 11152068

[B43] Guilloteau P Romé V Delaby L Mendy F Roger L Chayvialle JA A new role of phosphopeptides as bioactive peptides released during milk casein digestion in the young mammal: regulation of gastric secretion Peptides 2009 30 2221 2227 10.1016/j.peptides.2009.09.002 19744534

[B44] DeModena JA Gutiérrez S Velasco J Fernández FJ Fachini RA Galazzo JL Hughes DE Martín JF The production of cephalosporin C by *Acremonium chrysogenum *is improved by the intracellular expression of a bacterial hemoglobin Biotechnology 1993 11 926 929 10.1038/nbt0893-926 7763915

[B45] Hofmann G Diano A Nielsen J Recombinant bacterial hemoglobin alters metabolism of *Aspergillus niger* Metabol Engineer 2009 1 8 12 10.1016/j.ymben.2008.07.002 18694843

[B46] Suthar DH Chattoo BB Expression of Vitreoscilla hemoglobin enhances growth and levels of α-amylase in *Schwanniomyces occidentalis* Appl Microbiol Biotechnol 2006 72 94 102 10.1007/s00253-005-0237-x 16642333

[B47] Jeffery CJ "Moonlighting proteins" Trends Biochem Sci 1999 24 8 11 10.1016/S0968-0004(98)01335-8 10087914

[B48] Gancedo C Flores CL Moonlighting proteins in yeasts Microbiol Mol Biol Rev 2008 72 197 210 10.1128/MMBR.00036-07 18322039 PMC2268286

[B49] Jami MS García-Estrada C Barreiro C Abel-Alberto C Salehi-Najafabadi Z Martín JF The *Penicillium chrysogenum *extracellular proteome. Conversion from a food-rotting strain to a versatile cell factory for white biotechnology Mol Cell Proteomics 2010 9 2729 2744 10.1074/mcp.M110.001412 20823121 PMC3101859

[B50] Collinge AJ Trinci APJ Hyphal tips of wild-type and spreading colonial mutants of *Neurospora crassa* Arch Microbiol 1974 99 353 368 10.1007/BF00696249 4372967

[B51] Silverman-Gavrila LB Lew RR Calcium gradient dependence of *Neurospora crassa *hyphal growth Microbiology 2001 149 2475 2485 10.1099/mic.0.26302-0 12949173

[B52] Silverman-Gavrila LB Lew RR Regulation of the tip-high [Ca^2+^] gradient in growing hyphae of the fungus *Neurospora crassa* Eur J Cell Biol 2003 80 379 390 10.1078/0171-9335-00175 11484929

[B53] Gutiérrez S Velasco J Marcos AT Fernández FJ Fierro F Barredo JL Díez B Martín JF Expression of the *cefG *gene is limiting for cephalosporin biosynthesis in *Acremonium chrysogenum* Appl Microbiol Biotechnol 1997 48 606 614 10.1007/s002530051103 9421924

[B54] Sugano Y Nakano R Sasaki K Shoda M Efficient heterologous expression in *Aspergillus oryzae *of a unique dye-decolorizing peroxidase DyP, of *Geotrichum candidum* Appl Environ Microbiol 2000 66 1754 1758 10.1128/AEM.66.4.1754-1758.2000 10742277 PMC92058

[B55] Faus I Patiño C Río JL del Moral C Barroso HS Rubio V Expression of a synthetic gene encoding the sweet-tasting protein thaumatin in *Escherichia coli* Biochem Biophys Res Commun 1996 229 121 127 10.1006/bbrc.1996.1767 8954093

[B56] Chomczynski P Sacchi N Single-step method of RNA isolation by acid guaidinium thiocyanate-phenol-chloroform extraction Anal Biochem 1987 162 156 159 2440339 10.1006/abio.1987.9999

[B57] Sambrook J Russell DW Molecular Cloning: A Laboratory Manual 2001 Cold Spring Harbor Laboratory: Cold Spring Harbor, NY

[B58] Eymann C Becher D Bernhardt J Gronau K Klutzny A Hecker M Dynamics of protein phosphorylation on Ser/Thr/Tyr in *Bacillus subtilis* Proteomics 2007 7 3509 3526 10.1002/pmic.200700232 17726680

[B59] Jami MS Barreiro C García-Estrada C Martín JF Proteome analysis of the penicillin producer *Penicillium chrysogenum*: Characterization of protein changes during the industrial strain improvement Mol Cell Proteomics 2010 9 1182 1198 10.1074/mcp.M900327-MCP200 20154335 PMC2877979

[B60] Candiano G Bruschi M Musante L Santucci L Ghiggeri GM Carnemolla B Orecchia P Zardi L Righetti PG Blue silver: a very sensitive colloidal Coomassie G-250 staining for proteome analysis Electrophoresis 2004 25 1327 1333 10.1002/elps.200305844 15174055

[B61] Havlis J Thomas H Sebela M Shevchenko A Fast-response proteomics by accelerated in-gel digestion of proteins Anal Chem 2003 75 1300 1306 10.1021/ac026136s 12659189

