# Supplementary material for: Casein phosphopeptides drastically increase the secretion of extracellular proteins in Aspergillus awamori. Proteomics studies reveal changes in the secretory pathway
Source: Microb Cell Fact. 2012 Jan 10;11:5. doi: 10.1186/1475-2859-11-5 (PMC3283509; doi:10.1186/1475-2859-11-5)
Supplement: Additional file 1 — Additional Figure 1. Milk clotting assay for casein, casamino acids, NZ Amino A or phosphoserine. A. Serial dilutions (1/2) of a pure preparation of chymosin (500 ng) and the corresponding concentration in mg/L. B. Effect of casein, casamino acids, NZ Amino A and phosphoserine on chymosin production by strains TG-87 and TAPL-4. Culture supernatants from these strains were serially diluted from 1/2 to 1/512 (DF). No dilution is denoted as WD. The number of wells where milk clotting takes place (white precipitate) is proportional to the concentration of chymosin that is present in the culture supernatant. The chymosin concentration range that is present in those wells with clotting, has been estimated using the data obtained from panel A as it is indicated in the Methods section. [file 1475-2859-11-5-S1.PDF]

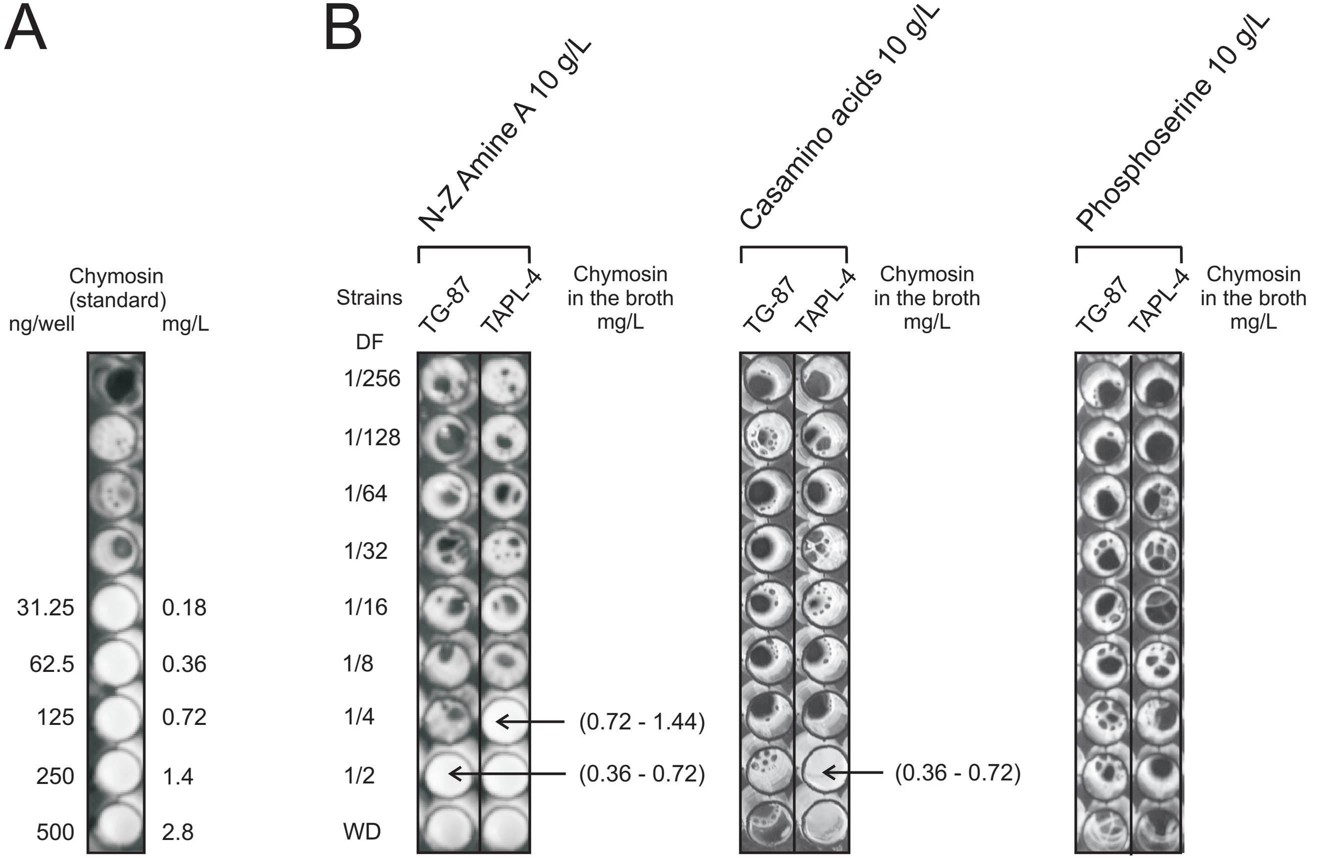

A
B
Chymosin (standard)
ng/well
mg/L
31.25
0.18
62.5
0.36
125
0.72
250
1.4
500
2.8
N-Z Amine A 10 g/L
Casamino acids 10 g/L
Phosphoserine 10 g/L
Strains
TG-87
TAPL-4
Chymosin in the broth mg/L
DF
1/256
1/128
1/64
1/32
1/16
1/8
1/4
1/2
WD
(0.72 - 1.44)
(0.36 - 0.72)
(0.36 - 0.72)
